# Integrative HLA typing of tumor and adjacent normal tissue can reveal insights into the tumor immune response

**DOI:** 10.1186/s12920-024-01808-8

**Published:** 2024-01-27

**Authors:** Angelina Sverchkova, Scott Burkholz, Reid Rubsamen, Richard Stratford, Trevor Clancy

**Affiliations:** 1 NEC OncoImmunity, Oslo Cancer Cluster, Innovation Park, Oslo, Norway; 2 Institute of Clinical Medicine, University of Oslo, Oslo, Norway; 3 Flow Pharma, Inc, Warrensville Heights, Galaxy Parkway, OH 4829 USA; 4 University Hospitals, Cleveland Medical Center, Cleveland, OH USA; 5 Case Western Reserve School of Medicine, Cleveland, OH USA

**Keywords:** HLA typing, Breast cancer, Immune cell infiltration, RNA-seq analysis

## Abstract

**Background:**

The HLA complex is the most polymorphic region of the human genome, and its improved characterization can help us understand the genetics of human disease as well as the interplay between cancer and the immune system. The main function of HLA genes is to recognize “non-self” antigens and to present them on the cell surface to T cells, which instigate an immune response toward infected or transformed cells. While sequence variation in the antigen-binding groove of HLA may modulate the repertoire of immunogenic antigens presented to T cells, alterations in HLA expression can significantly influence the immune response to pathogens and cancer.

**Methods:**

RNA sequencing was used here to accurately genotype the HLA region and quantify and compare the level of allele-specific HLA expression in tumors and patient-matched adjacent normal tissue. The computational approach utilized in the study types classical and non-classical Class I and Class II HLA alleles from RNA-seq while simultaneously quantifying allele-specific or personalized HLA expression. The strategy also uses RNA-seq data to infer immune cell infiltration into tumors and the corresponding immune cell composition of matched normal tissue, to reveal potential insights related to T cell and NK cell interactions with tumor HLA alleles.

**Results:**

The genotyping method outperforms existing RNA-seq-based HLA typing tools for Class II HLA genotyping. Further, we demonstrate its potential for studying tumor-immune interactions by applying the method to tumor samples from two different subtypes of breast cancer and their matched normal breast tissue controls.

**Conclusions:**

The integrative RNA-seq-based HLA typing approach described in the study, coupled with HLA expression analysis, neoantigen prediction and immune cell infiltration, may help increase our understanding of the interplay between a patient’s tumor and immune system; and provide further insights into the immune mechanisms that determine a positive or negative outcome following treatment with immunotherapy such as checkpoint blockade.

**Supplementary Information:**

The online version contains supplementary material available at 10.1186/s12920-024-01808-8.

## Introduction

The Major Histocompatibility Complex (MHC) is a dense gene cluster found in vertebrates. It plays a crucial role in the immune response by presenting foreign or aberrated antigens to T cells [1]. The MHC complex in humans is known as the Human Leukocyte Antigen (HLA) complex. Its molecules are encoded by genes located on the short arm of chromosome 6 (6p21.3) [2]. The classical HLA Class I molecules (*HLA-A*, *-B,* and *-C*) are found on the surface of all nucleated cells and present endogenous antigens originating from the cytoplasm to cytotoxic T cells. Classical HLA Class II molecules (*HLA-DP*, *-DQ,* and *-DR*) are mainly found on antigen-presenting cells such as macrophages, dendritic cells, and B cells, and present exogenous antigens extracellularly from foreign bodies such as pathogens to helper T cells [3]. The HLA region plays an important role in infectious diseases and autoimmunity [4–6], tumor development [7–9], organ transplantation [10], and drug hypersensitivity [11]. The complex also contains non-classical HLA genes, including Class I *HLA-E*, *HLA-F* and *HLA-G*, and Class II *HLA-DOA*, *HLA-DOB* and *HLA-DM*. These non-classical alleles have a broad range of functions in the antigen-presenting pathway and their alteration has been previously shown to provide tumor cells with different mechanisms for evasion from host immune surveillance resulting in tumor growth [12].

The HLA complex is the most polymorphic gene region in the human genome [13]. Its high sequence diversity can be explained by the need to successfully display a wide range of processed foreign peptides to the T cell antigen receptor [14–16]. In fact, it has been shown that populations from pathogen-rich geographic regions exhibit increased HLA diversity in relation to their average genomic diversity [14] and heterozygous HLA individuals display enhanced pathogen resistance compared to HLA homozygotes [17], as well as increased efficacy toward cancer immunotherapies [18]. Currently, over 26,000 Class I and 11,000 Class II alleles have been characterized and reported in the IPD-IMGT/HLA database (DB) – a specialist DB for sequences of the HLA complex (IPD-IMGT/HLA release 3.54; October 2023) [19]. This large number of alleles has led to the development of special nomenclature whereby each HLA allele has a unique name corresponding to up to four sets of digits separated by colons (e.g., HLA-A*01:01:01:01). The first set of digits corresponds to the serological antigen carried by an allotype; the second to the protein type; followed by the third set of digits corresponding to synonymous changes in coding regions; and the last to the changes in non-coding regions [20].

Capturing the specific allelic combinations in individuals, or HLA typing, has important implications in diverse areas including organ and stem cell transplantation [10, 21], disease association studies [5], drug safety [22] and neoantigen prediction for the development of personalized cancer immunotherapies [23, 24]. HLA typing with two fields of resolution (HLA indexed with two sets of digits separated by a colon, e.g., HLA-A*01:01) is often used for clinical purposes, as it defines the specific HLA allele at the protein-coding level. Even a single amino acid difference between two HLA proteins of the same antigen group (same first set of digits) can result in altered T cell recognition specificity and allograft rejection [25]. Multiple laboratory-based techniques for HLA typing have been established including sequence-specific oligonucleotide probe (SSOP), sequence-specific primer (SSP), and sequence-based typing (SBT) [26]. These methods allow accurate high-resolution HLA typing but remain labour intensive and time-consuming, expensive, and low throughput [27]. In recent years targeted next-generation sequencing (NGS)-based methods have become a new gold standard in the HLA typing field as they increase throughput while decreasing labour costs [28]. However, despite these advantages, targeted NGS techniques have some limitations, as they only profile the HLA region and consequently may miss useful information that is situated in other regions of the genome. Recent advancements in NGS technologies have significantly impacted the HLA typing process [29]. HLA genotyping from standard short-read data is practical as it allows massive, parallel, high-resolution HLA typing. However, whole-genome or exome sequencing often employs a reduced read length and coverage compared to targeted sequencing, which makes it challenging to resolve highly homologous alleles that may differ by a single nucleotide. Furthermore, HLA typing from NGS is complicated by the existence of HLA pseudogenes which are very similar to classical HLA alleles leading to incorrect mapping of reads [30]. HLA typing from RNA sequencing is further complicated by post-transcriptional modifications and bias due to amplification [31]. However, RNA-seq-based HLA typing presents a distinct advantage over DNA-based methods as it allows for the simultaneous HLA expression estimation and HLA genotyping from the same patient sample.

Given the wide availability and accessibility of RNA-seq and the importance of HLA expression in mediating an immune response, developing techniques for HLA typing from RNA could be of great value. Moreover, since RNA-seq is widely used in the precision oncology [32] it would be convenient and cost-effective to predict HLA haplotypes directly from RNA-seq, potentially avoiding the need for additional tests.

While multiple algorithms for HLA typing from RNA-seq have been developed over the last decade [33–42], many deliver ambiguous predictions, while others claim to have accuracies that could not be reproduced in other studies [43]. In addition, the majority of these methods, including OptiType [36], HLAminer [34] and Seq2HLA [33], limit their reference sequences to core exons responsible for antigen binding affinity (exons 2 and 3 in HLA Class I and exon 2 in HLA Class II loci), making it difficult or impossible to accurately type/deconvolute certain alleles, or discover novel or uncharacterized alleles [44].

Here we present an advance on our previous *in-silico* HLA typing method from DNA data, OncoHLA [45, 46], which now captures both Class I and II genotypes from RNA-seq reads. We evaluated the performance of the method on a gold-standard benchmarking RNA-seq dataset [47] for HLA typing against five previously published algorithms, including OptiType [36], Seq2HLA [33], HLAProfiler [41], HLApers [42] and ArcasHLA [39]. We demonstrate here that OncoHLA performs on par with the state-of-the-art HLA Class I typing tools, and outperforms the state-of-the-art tools when considering both Class I and Class II combined (using RNA-seq data).

In addition to the HLA genotype itself, diversity in HLA allelic expression may also influence immune responses. Besides HLA typing from RNA-seq, the strategy we describe here is adapted to infer personalized (allele-specific) HLA transcript abundance. The integrative HLA typing from DNA, RNA, and the subsequent quantification of the typed HLA in tissue may be an important guide in clinical studies. This is especially important in the context of studying the immune response to cancer. Tumors can evolve diverse mechanisms to escape T cell recognition [48]. The downregulation of HLA expression in tumor cells results in decreased presentation of tumor antigens by HLA on the cell surface and has been shown to significantly impact patient prognosis [9, 49, 50]. Moreover, total HLA Class I antigen loss due to the mutation of *beta-2 microglobulin* (*B2M*) – a component of HLA Class I molecules, has been found in several types of cancer [51–54]. Analysis of matched tumor and normal profiles may be crucial to improve our understanding of tumor escape mechanisms and response (or lack of it) to immunotherapy. However, RNA-seq data from tumor tissue is not usually matched with RNA-seq data from its normal tissue counterpart, as this tissue material is often not accessible or available to excise from a biopsy or surgical resection. This practical constraint may limit our understanding of the role that HLA expression plays in determining tumor immune escape and its impact on patient response to therapy, as the context of its expression relative to the normal tissue counterpart cannot be considered. Here, to demonstrate the utility of “integrative HLA typing”, we applied our strategy on two independent datasets where primary breast cancer and matched adjacent normal breast tissue were available (consisting of triple-negative breast cancer (TNBC) and estrogen receptor-positive (ER +)/HER2-negative (HER2-) tumors). We demonstrate that integrative HLA typing that incorporates an investigation of differential personalized HLA expression between tumor and matched normal adjacent tissue can shed light on the interaction between the host immune system and tumor and provide insights into the anti-tumor immune response.

## Materials and methods

### Database construction / HLA reference sequences

We downloaded a file written in Extensive Markup Language (XML) from IPD-IMGT/HLA DB version 3.46.0. The XML format combines the data included in the sequence alignments with the data available in the individual allele reports. OncoHLA’s reference library for typing from RNA-seq reads was constructed from all exons. However, many alleles in the DB had incomplete sequences, containing in many cases only sequences covering exons encoding for the peptide-binding site as they contain most of the polymorphism. In case if some exons were not covered in the DB, we reconstructed them by taking the sequence from the closest allele containing the necessary information. The closest allele is the one that has the highest sequence identity with the allele with missing sequence information. In cases where multiple alleles with the same sequence identity were present, the choice was made in favor of confirmed and common, well-documented alleles.

### Genotyping algorithm

The basis of the algorithm has been described in the previous paper for typing from DNAseq reads [45]. The method uses integer linear programming algorithm (ILP) that searches for the optimal combination of alleles from all loci that maximizes the number of reads potentially originated from this selection. The only difference in the case of RNA-seq typing is that it uses one-step ILP instead of two. In the case of typing from WES and WGS, the library includes introns and therefore longer sequences which makes it slow to type from all known complete sequences at once. First, the algorithm searches for the best candidates relying on exons coding for peptide binding domain and flanking introns, and then relies on the complete sequences of the alleles to resolve ambiguities and choose one or two candidates per locus depending on its zygosity status. In the case of typing from RNA-seq, the reference sequences do not contain introns making the typing less complex computationally, and therefore one step can be used for genotyping.

### Validation data

To evaluate the performance of OncoHLA on RNA-seq data against previously published tools, a publicly available dataset of RNA sequencing data by the GEUVADIS (Genetic European Variation in Health and Disease) consortium was used [47]. The data contains 462 human lymphoblastic cell line samples from 5 different populations: CEU, FIN, GBR, TSI and YRI populations from the 1000 Genomes sample collection. The dataset has been used for benchmarking by several previously published tools and is considered to be the gold standard dataset for RNA-seq HLA typing evaluation.

The data that passed quality control were downloaded in bam format from: http://www.ebi.ac.uk/arrayexpress/experiments/E-GEUV-1/files/processed. Reads potentially originating from the HLA region were extracted, these include all reads from chromosome 6 that are mapped in a proper pair, all pairs where one read is mapped to chromosome 6 with an unmapped pair, and all pairs where both reads are unmapped.

HLA types for 358 of these samples were determined using Sanger sequences for all three Class I loci and 2 Class II loci (*HLA-DRB1* and *HLA-DQB1*) [55]. Only the peptide-binding region for each gene was sequenced. The typing relied on IPD-IMGT/HLA database from 2009. We used the list of ambiguous allele combinations provided by IPD-IMGT/HLA to evaluate the performance of OncoHLA and other tools.

### Benchmarking set: choice of previously published tools for comparison purpose

To evaluate the performance of the developed method and compare it with the existing algorithms we ran OncoHLA together with previously published tools on GEUVADIS dataset which is publicly available. The choice of the tools was mostly based on previously published benchmarking results. We have chosen five tools: Seq2HLA [33], OptiType [36], HLAPers [42], HLAProfiler [41], and ArcasHLA [39] since they outperform other tools according to recent publications [39, 43]. All the methods have already been run on this dataset before in several studies, but the inferred accuracies differ from one study to another. Therefore, we have decided to run all the tools ourselves using the same input and default parameters.

### Configuration of the tools from the benchmarking set

- OptiType was downloaded from https://github.com/FRED-2/OptiType and ran with default parameters using the following command:
  OptiType -i [fastq_1] [fastq_2] -r -o [output_dir]
  The reference library used by OptiType was updated in 2014 and has not been updated since that time.
- Seq2HLA was downloaded from https://github.com/TRON-Bioinformatics/seq2HLA and ran using the command:
  python seq2HLA.py -1 [fastq1] -2 [fastq2] -r [run_name] -p 10
  The reference library goes together with the tool and was updated in 2017.
- HLAProfiler was downloaded from https://expressionanalysis.github.io/HLAProfiler/ and ran with the default parameters using the command:
  perl HLAProfiler.pl predict -fastq1 [fastq_1] -fastq2 [fastq_2] -database_name hla_database -database_dir HLAProfiler-1.0.0-db_only -reference HLAProfiler-1.0.0-db_only/hla_database/data/reference/hla.ref.merged.fa -output_dir [output_dir] -kraken_path kraken-0.10.5-beta-ea.1 -if -l [sample_name].HLAProfiler.log
  The database was updated in 2017 and was downloaded from:
  https://github.com/ExpressionAnalysis/HLAProfiler/releases/tag/v1.0.0-db_only
- HLApers was downloaded from https://github.com/genevol-usp/HLApers and ran with Kallisto [56] and the library made from IPD-IMGT/HLA version 3.48.0 with the following command:
  genotype -i HLApers/index -t HLApers/hladb/transcripts_MHC_HLAsupp.fa -1 [fq_file1] -2 [fq_file2] -o [outprefix_name] –kallisto6
- ArcasHLA was downloaded from https://github.com/RabadanLab/arcasHLAand and ran with the library made of complete alleles from IPD-IMGT/HLA version 3.46.0 using the command:
  arcasHLA genotype [fastq_1] [fastq_2] -g A,B,C,DRB1,DQB1 -o [output_dir]

All the tools were run using the same fastq files as OncoHLA obtained by the procedure described in ‘Validation data’ paragraph.

### Prediction accuracy / performance measure

Predictions were considered accurate if a predicted allele matched any allele in the G group of the experimentally detected allele. HLA alleles that have identical nucleotide sequences across the exons encoding the peptide binding domains (exon 2 and 3 for HLA class I and exon 2 only for HLA class II alleles) are part of the same G group which is named by the first three fields of the lowest numbered allele in the group followed by ‘G’. This list was obtained from the IPD-IMGT/HLA DB under the name ‘hla_nom_g.txt’.

### Allele-specific expression quantification

We quantified abundances of HLA transcripts from RNA-seq data using Kallisto [56]. To obtain reliable expression levels for each sample, we used inferred HLA genotypes as a transcriptome index. This requires an additional step consisting in removing all HLA transcripts from human reference transcriptome file and adding the sequences of a person’s own HLA alleles. We used transcript per million (TPM) as the measurement of HLA abundances.

### Immune cells fraction quantification

To estimate the fraction of different immune cells, we used quanTIseq tool [57]. It quantifies the proportions of ten different immune cell types via deconvolution. We used the output of Kallisto with personalized HLA abundances to calculate the gene TPMs by summing all the abundances of the transcripts which constituted the gene. The inferred gene TPMs were used as an input to quanTIseq.

### Breast cancer data

We applied the RNA-seq typing approach on two independent RNA-seq datasets. The first dataset is publicly available and contains 42 triple negative breast cancer (TNBC) and 42 ER + /HER2- primary tumors as well as 21 uninvolved breast tissue samples that were adjacent to TNBC tumors and 30 uninvolved breast tissue samples that were near ER + breast tumors [58]. The samples were sequenced on Illumina HiSeq 2000 sequencing machine (2 × 50 bp) and are available under accession number GSE58135. 17 samples that had most of their reads removed after trimming and quality control of the sequencing were discarded from the analysis. We matched the left tumor–adjacent normal samples using HLA genotypes inferred by our method resulting in 20 ER + -normal pairs and 15 TNBC normal pairs (Supplementary Table 1). The second dataset is in-house and cannot be made publicly available because of privacy concerns. It consists of 5 ER + /HER2-patients for which WES and RNA-seq were available including both primary tumor and adjacent normal tissues. Tumor identification was made in the operating room via palpation or radio-guided biopsy markers. The tumor was excised, marked for orientation, and sent for immediate pathological tissue handling for the study. A small piece of normal breast tissue was obtained from the surgical site, away from the tumor area, or from the medial aspect of the sentinel lymph node incision. Normal breast tissue samples were collected prior to tumor excision to minimize potential cross-contamination with the tumor tissue.

The identities of tissues, either breast tumors or adjacent normal tissues, were confirmed by a pathologist using standard Hematoxylin and Eosin (H&E) staining of parallel frozen sections and formaldehyde-fixed paraffin-embedded (FFPE) tissue blocks. The plane of the tumor tissue sample taken for gene sequencing was as close as possible to the tumor plane sent for permanent blocks so that the H&E slide images would represent the sequenced region as closely as possible.

Tissue that was not processed for pathology was dissected to produce approximately 100 mg pieces with dimensions less than 0.5 cm. These samples were snap-frozen in tubes capable of withstanding cryopreservation and labeled only with the patient’s unique code for that replicate. Tumor tissue samples were collected for DNA and RNA analyses, whereas normal tissue samples were collected for RNA analysis only.

DNA and RNA were extracted from the respective samples using the QIAGEN reagent (QIAzol, QIAGEN, Hilden, Germany). Whole-exome sequencing was performed on the tumor and normal tissues. Sequencing libraries were generated from the extracted DNA using the Agilent SureSelectXT2 Human All Exon V6 Kit (Agilent Technologies, Santa Clara, CA, USA). Normal and tumor tissue samples were sequenced at 100 × and 300 × coverage, respectively. RNA sequencing was performed on the tumor and normal breast tissues to profile the transcriptome. Messenger RNA was captured via poly-A tails and prepared for sequencing using NEBNext Ultra II (New England Biolabs Inc., Ipswich, MA, USA). An Agilent 2100 bioanalyzer (Agilent, Santa Clara, CA) was used to process samples, report the RNA integrity number (RIN), and visualize the ribosomal ratios. All RIN scores from the passing samples were above 6.0, with most samples scoring above 7.5. Normal samples were sequenced at 60 million paired-end reads and tumor samples were sequenced at 120 million paired-end reads. NGS was performed on all samples using NovaSeq6000 (Illumina, San Diego, CA, USA) with paired-end reads of 150 bp.

### Antigen-presenting score

The antigen presentation of tumor-specific mutations was assessed using the NEC Immune Profiler (NIP) software from NEC OncoImmunity, comprising of several proprietary machine-learning (ML) prediction algorithms. The algorithm considers the following features when predicting the immunogenicity of a candidate:

1. The binding affinity of the mutated peptide to HLA.
2. The peptide’s ability to be efficiently processed by the antigen processing machinery (APM).
3. The expression of the candidate neoantigen.
4. The ability of the somatic mutation’s host protein to harbor HLA bound presented peptides.

Class I neoantigen predictions were conducted for each of the 5 ER + /HER2- patients for which WES and RNA-seq were available including both primary tumor and adjacent normal. The publicly available dataset comprised RNA-seq data only and was therefore unsuitable for the neoantigen predictions.

### Statistical analysis

HLA gene expression and immune cell infiltrations comparison between primary tumor and corresponding normal tissue was performed using Student’s t-test. Differences were considered statistically significant when *P*-values < 0.05. In addition, Bonferroni correction for multiple comparisons was also applied and adjusted *P*-values were calculated (Supplementary Tables S3 and S4).

## Results

### Performance and benchmarking with other tools

**Fig. 1:**
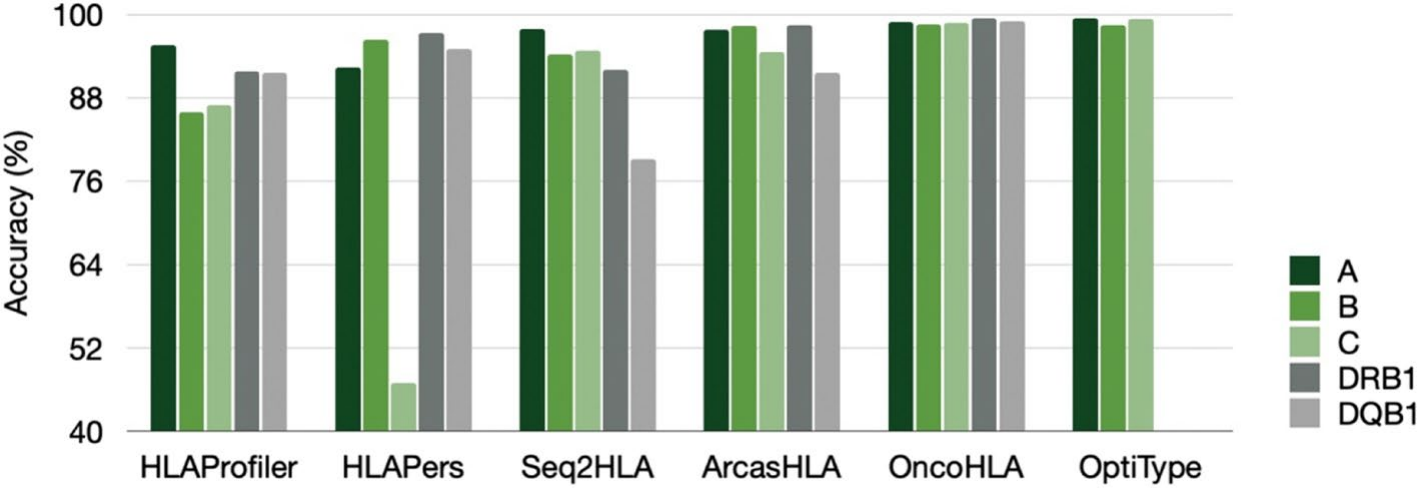
Figure showing HLA typing accuracies for OncoHLA and five previously published tools on 358 GEUVADIS samples with experimentally detected HLA genotypes. Accuracies representing the fraction of alleles correctly called are shown as a percentage for each locus separately and were calculated at two fields of resolution

The GEUVADIS dataset has been used by many RNA-seq-based HLA typing methods and is considered a gold standard dataset for the evaluation of HLA genotyping from RNA-seq reads. The accuracy with two-field resolution for OncoHLA applied to RNA-seq data, and five other RNA-seq HLA typing tools was assessed and is shown in the Fig. 1. The performance for each locus was measured separately. OncoHLA showed an overall accuracy of 98.74% at Class I and 99.23% at Class II at high-resolution outperforming Seq2HLA, HLAProfiler, HLApers and ArcasHLA in both MHC Classes. OptiType showed an accuracy of 99.07% at Class I which is 0.33% higher than our method. OptiType uses an old version of IPD-IMGT/HLA DB dated to 2014 with a significantly lower number of alleles compared to the contemporary versions of the DB and considers only those alleles that were reported in allelefrequencies.net [59] or dbMHC [60]. Moreover, OptiType’s HLA library includes only exons coding for the peptide-binding site and flanking exons, bringing it closer to the settings of the experimental typing and reducing noise by having a restraining number of alleles in its reference HLA library. For Class II, the second-best result was obtained by ArcasHLA which had an accuracy of 95.04%, which is 4.19% less compared to OncoHLA. We could not reproduce the exact performance stated in the original publications of the previously published algorithms, except for OptiType.

Buchkovich et al., have re-sequenced and updated the ground truth HLA genotype of 34 GEUVADIS samples having at least one allele that was discordant between Sanger sequencing and either OptiType or HLAProfiler [41]. We have also compared the performance of the benchmarking set using the updated reference and the results are shown in the Supplementary Table 2. OptiType showed an accuracy of 99.7% for Class I, followed by OncoHLA which showed an accuracy of 99.2% for Class I and 99.8% accuracy for Class II genes. The next highest performance on HLA Class II typing of 96.8% accuracy was reached by HLApers.

### Matching tumor and normal samples

The publicly available dataset containing RNA-seq performed on two different subtypes of breast cancer (TNBC and ER + /HER2-) and histologically normal adjacent tissues was first processed to match tumor with normal breast samples as this information was not available in the publication. To achieve this, we compared all the HLA genotypes obtained with our method. Even though all loci were used to perform the matching, we noticed that genotypes of classical Class I alleles were sufficient to accurately match the tumor-normal pairs. After this processing step, we obtained 20 ER + -normal and 15 TNBC-normal pairs. All the genotypes between tumor and normal samples were identical at the nucleotide level in the coding region (3 fields of resolution). Accession numbers of matched pairs as well as predicted genotypes for classical HLA Class I genes are shown in the Supplementary Table 1.

### TNBC and ER + tumors can be characterized by varied tumor vs. normal HLA expression

**Fig. 2:**
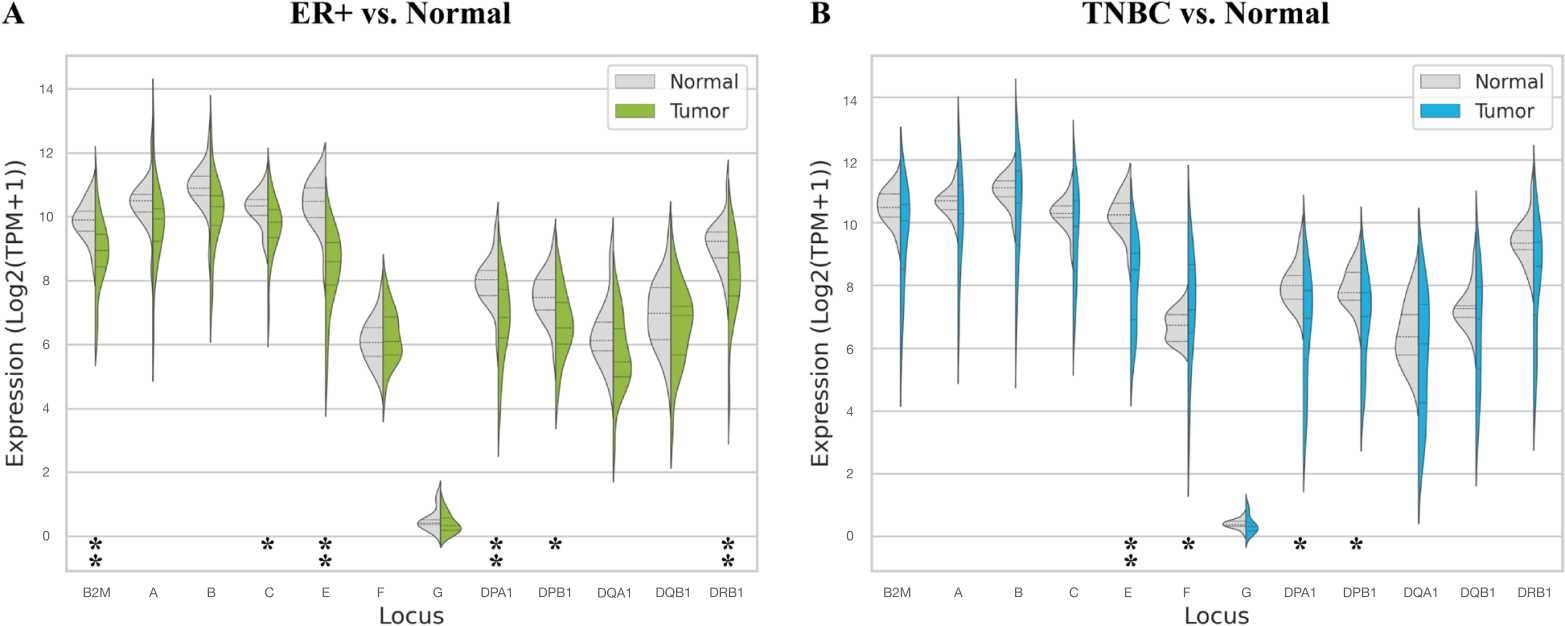
Violin plots representing log-scaled allele-specific expression of classical and non-classical HLA loci and B2M protein in two breast cancer subtypes (in green for ER + /HER2- and blue for TNBC subtypes) and normal adjacent tissues (in gray). **A** ER + /HER2- primary tumor vs. matched normal adjacent tissue. **B** TNBC primary tumor vs. matched normal adjacent tissue. The dotted lines represent the quartile positions. * *P*-value < 0.05, ** *P*-value < 0.01

We investigated the differential expression of HLA and *B2M* across two different subtypes of breast cancer including the ER + /HER2- and TNBC primary tumors from the publicly available dataset. Increased variation in HLA and *B2M* expression was observed in both breast cancer subtypes compared to normal adjacent tissues (Fig. 2, Supplementary Figure S1). TNBC tumors depicted a higher variation of HLA and *B2M* expression compared to ER + tumors. We found that the medium expression of *B2M* and most of the HLA alleles decreased in both cancer subtypes compared to adjacent normal tissues. Statistical analysis showed that *B2M*, which forms the small light chain subunit of the MHC Class I molecules and plays a crucial role in antigen presentation, was downregulated in ER + breast cancer, but was not significantly different in TNBC subtype compared to matched normal samples. In addition, *HLA-C*, *-E*, *-DPA1*, *-DPB1*, *-DRB1* were also significantly downregulated in ER + . As for TNBC, the *HLA-E*, *-DPA1*, *-DPB1* alleles were significantly downregulated, while *HLA-F* was significantly upregulated. No significant change in *HLA-F* expression was found in ER + primary tumors. Interestingly, after applying more strict statistical testing on the differential expression of HLA genes, whereby we adjusted the *P*-values with Bonferroni multiple correction and performed filtering based on fold change, HLA-E maintained a significant downregulation in both breast cancer subtypes compared to their normal tissue counterparts (Supplementary Tables S2 and S4, Supplementary Figure S2).

### Decreased HLA-E expression in breast cancers corresponds with increased NK cell infiltration

**Fig. 3:**
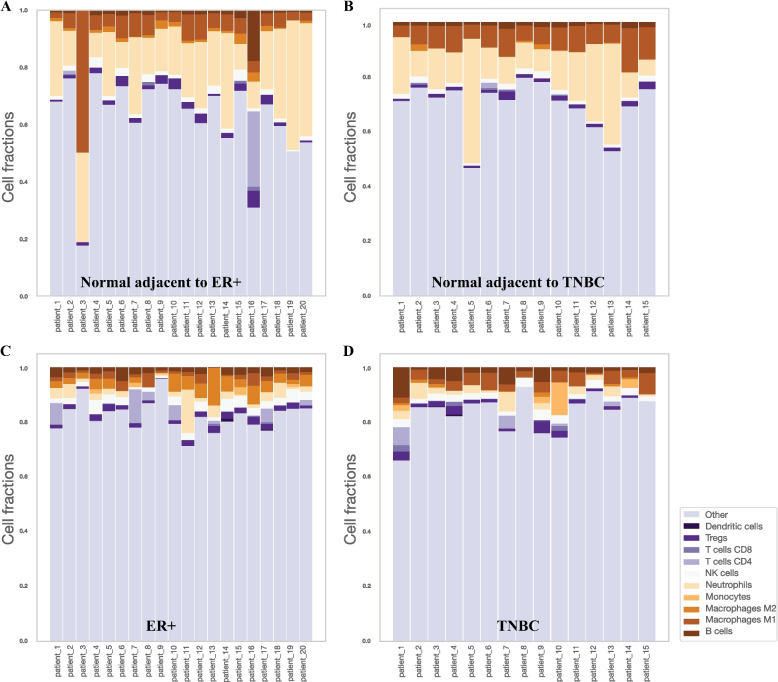
Bar plots representing the predicted immune cell fractions. **A** Immune cell fractions in normal adjacent to tumor tissues of 20 patients with ER + /HER2- breast cancer. **B** Immune cell fractions in normal adjacent to tumor tissues of 15 patients with TNBC. **C** Immune cell fractions in primary tumors of 20 patients with ER + /HER2- breast cancer. **D** Immune cell fractions in primary tumors of 15 patients with TNBC

As both *HLA-E* and *HLA-F* have been shown to be implicated in the mediation of immune evasion and immune suppression by inhibiting cells of the adaptive and innate immune system, we attempted to compare the absolute proportion of different immune cell phenotypes between breast cancer and adjacent normal tissues (Fig. 3) [61, 62]. After comparing the fractions of 10 different immune cells (B cells, Macrophages M1, Macrophages M2, Monocytes, Neutrophils, Natural Killer (NK) cells, T cells CD4, T cells CD8, Tregs and dendritic cells) between tumors and matched histologically normal tissues, we found that the fractions of NK cells were significantly increased in both breast cancer subtypes. It is known that *HLA-E* inhibits NK cell-mediated lysis by interacting with CD94/NKG2A receptors [63]. Therefore, the downregulation of *HLA-E* may induce the observed increase in NK cell infiltration. NK cells play a crucial role in the nonadaptive immune system and kill target cells that exhibit a reduced abundance of surface-bound Class I HLA molecules. They express receptors that interact with MHC Class I serving to inhibit cell-mediated cytotoxicity [64]. At the same time, *HLA-F* molecules can interact with both activating and inhibitory receptors on NK cells, in particular, open conformers of *HLA-F* are high-affinity ligands of the activating NK-cell receptor KIR3DS1 [65]. Tumors can deploy different mechanisms to escape from NK cells and differential expression of *HLA-E* and *HLA-F* may act synergistically to promote tumor survival. This suggests that the expression of non-classical HLA Class I molecules, which plays an important role in mediating immune surveillance by NK cells, may modulate the breast tumor immune cell interaction and potentially mediate immune escape. In addition, an increased fraction of B cell infiltration was observed in TNBC tumors exclusively which is consistent with previous studies showing that tumor-infiltrating B cells are more frequently observed in the TNBC and HER2 + subtypes [66]. Moreover, a potential immunosuppressive environment was observed by a significant predicted decrease in M1 macrophages and an increase in M2 macrophages in both breast cancer subtypes.

### Validation on an independent dataset of ER + matched normal tissue pairs provides insights into breast cancer immunology

To further investigate the correspondence between our personalized integrative HLA typing approach, HLA expression quantification in tumor vs. normal, and the subsequent impact on the immunobiology of tumors, we applied our integrative approach on five ER + /HER2- primary breast tumors with matched histologically normal adjacent breast tissue. The five tumor samples and their matched normal adjacent tissues were sequenced both at the DNA level using whole exome sequencing (WES) and HLA typed using the previously published version of OncoHLA [45], and at the RNA level with the HLA typing performed using the RNA-seq HLA typing extension discussed in this study. No discordances were found between HLA typing from WES and RNA-seq at two fields of resolution, however, one disagreement at the third field of resolution was observed in the DQA1 locus in patient 4. This corresponds to a 100% match between HLA typing from WES and RNA-seq at two fields of resolution and a 98.75% match at three fields of resolution. As for the fourth field of resolution corresponding to changes in the non-coding region, 2 discordances were detected between WES tumor and WES matched normal in patient 2 in the DPB1 and the DQA1 loci.

**Fig. 4:**
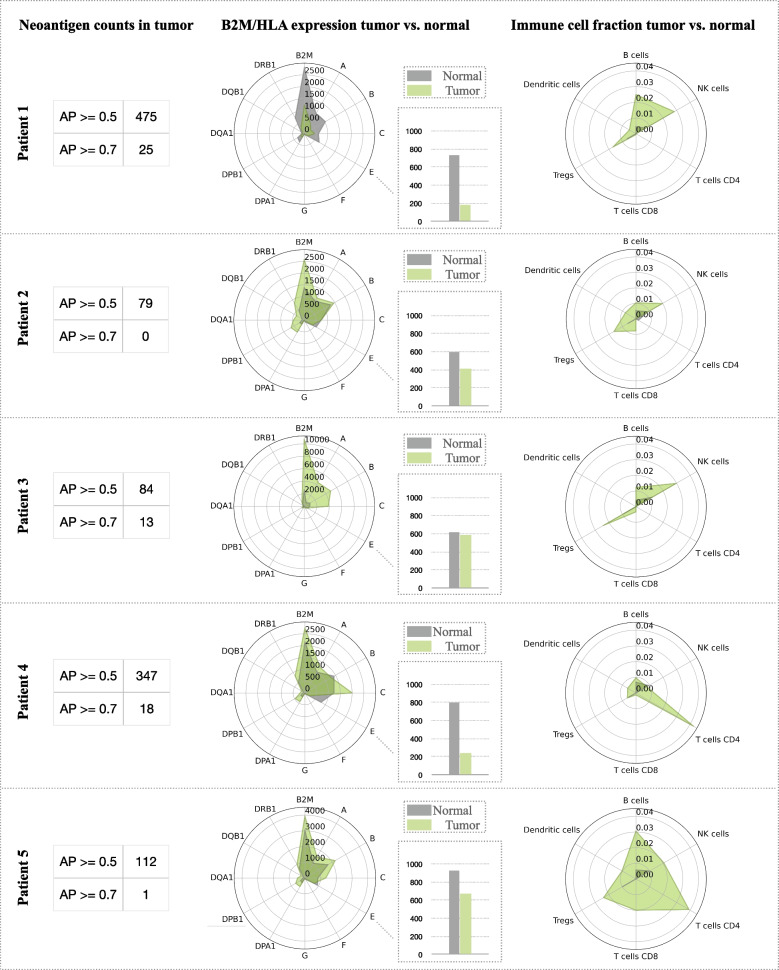
Figure representing in-depth analysis of 5 patients with ER + /HER2- breast cancer. The antigen counts in tumor with antigen-presenting (AP) scores >  = 0.5 and >  = 0.7 are shown in the tables on the left side of the figure. The AP scores >  = 0.5 and >  = 0.7 are indicative of an increased likelihood of presenting the candidate neoantigen peptides. Radar plots in the middle of the figure display personalized HLA/B2M expression in tumor (green) versus matched normal adjacent tissues (gray). The numbers on the axes correspond to the TPM values of the HLA genes. The relative position of axes and the angles between the axes represent no biological meaning. HLA-E expression is shown in bar plots in the footnote of each radar plot. The radar plots on the right show immune cell fractions for tumor (green) and normal adjacent to tumor tissues (gray)

In Fig. 4 we provide an in-depth patient-specific breakdown of the induvial HLA expression results in the tumor versus normal, and the subsequent impact on the immune microenvironment. While the small sample size prohibited rigorous hypothesis testing, the data did reveal some interesting and insightful trends. For example, similarly to the publicly available breast cancer dataset, we observed a decrease in *HLA-E* expression in the proprietary ER + /HER2- breast tumors compared to the normal adjacent to tumor tissue, and an increased infiltration of NK cells in four out of five patients (Fig. 4). NK cell infiltration was decreased marginally in patient 4, however, this was accompanied by a relatively high predicted neoantigen burden alluding to an abrogation of the need for NK cell tumor infiltration in that specific case. The increased expression of classical HLA Class I alleles that typically present peptide antigens for T cell recognition was observed in most tumors, except for patient 1. However, in patient 1, the decreased expression of the peptide presenting HLA alleles (*HLA-A*, *-B*, and -*C*) and *HLA-E* was accompanied by a notably increased infiltration of NK cells into the tumor. It is tempting to speculate that the tumor cells harboring a relatively high neoantigen load in patient 1 avoid killing by T cells, as these tumor cells may not present neoantigens due to the downregulation of classical Class I HLA alleles.

## Discussion and conclusions

The HLA complex is an integral part of the human immune system and is mandatory for immune cell recognition and subsequent tumor cell killing. Alterations in HLA expression are often used by tumors as a mechanism of immune escape inducing an immunosuppressive environment [9, 67–70]. The development of cancer therapies must arguably consider these HLA-associated immune escaping mechanisms as they have been shown to occur frequently in the majority of cancer types [71]. RNA-seq is a widely used approach for transcriptome profiling in biomedical research and can be used to perform both high throughput HLA genotyping and estimation of HLA expression levels. Conventional RNA-seq analyses often involve read alignment to a reference genome with subsequent reconstruction of the transcriptome and calculation of gene abundance. However, the polymorphic nature of the HLA complex is not reflected in the reference genome preventing accurate read alignment resulting in incorrect expression estimation of HLA alleles. Given the importance of HLA expression in possible tumor immune escape, it is important to consider the polymorphism of the HLA complex and perform personalized HLA expression analysis. Numerous computational HLA typing methods applied to RNA-seq data have been published [34–36, 39, 41, 72], however, only a small subset of these provide allele-specific or personalized HLA expression estimation [33, 42].

The aim of this study was to develop an integrated approach for analyzing the HLA complex using RNA-seq data that genotypes HLA alleles and infers personalized HLA expression at the allelic level, and show its potential in studying the interaction between cancer and the host immune system, using RNA-seq data from tumors and their normal adjacent tissues. We demonstrated that our method for HLA typing from RNA-seq achieved an accuracy above 99% outperforming the previously published tools that can type both Class I and Class II HLA genes. Our study of primary breast cancer and normal adjacent tissue demonstrated that an integrative approach studying personalized HLA expression, neoantigens and immune cell infiltration may improve our understanding of the interplay between a tumor and the patient’s immune system. Moreover, our analyses of tumor vs. normal HLA genotype expression indicate that the relative expression of non-classical HLA alleles (that may serve as activating or inhibiting ligands to NK cells) may play an important role in governing tumor-immune interactions, in addition to the expression levels of the classical antigen-presenting HLA. We observed a down-regulation of non-classical *HLA-E*, an NK cell inhibitory ligand [64], corresponding to increased NK cell infiltration in breast tumors compared to adjacent normal tissues, in both the TNBC and ER + /HER2- cancer subtypes. Further experimental studies may be required to shed light on the connection between these two phenomena. Moreover, we have found that the non-classical *HLA-F* allele was upregulated in TNBC and had no significant change of expression in ER + /HER2- tumors. The biological function and clinical implications of altered *HLA-E* expression have been better studied than those of *HLA-F* [73]. Previously, upregulation of *HLA-F* has been found to be associated with poor survival in cancer patients [74, 75]. Triple-negative breast cancer patients have the worst prognosis and are considered the patients of choice for immunotherapies [76]. Therefore, the function of the *HLA-F* NK cell axis as an important immune regulatory interaction may be an important angle for further studies in the context of TNBC.

The dysregulation of HLA expression has been observed in many different types of cancer including melanoma, lung, breast, and prostate cancer [8, 77–79]. Chowell et al., demonstrated that physiochemical sequence divergence between HLA Class I alleles of patient’s genotypes influences their response to immune checkpoint inhibitor treatments [18]. This discovery highlights the importance of HLA genotyping in cancer research and treatment. However, currently, there is not a bulk of published evidence characterizing the role of HLA expression as a potential biomarker for patient stratification or for predicting tumor responses to immunotherapies. Nevertheless, some initial advances have been already made in this area [80]. For example, Rodig et al., demonstrated that HLA Class I and Class II expression can serve as a reliable predictive biomarker of tumor response to a group of immune checkpoint inhibitor-based immunotherapies [81]. Also, it has been shown that the expression level of a non-classical HLA-G gene is correlated with different clinical parameters in many tumors [82]. For example, the high expression of this gene in hepatocellular carcinoma has been shown to be associated with poor outcome [83]. Given the crucial role that HLA molecules play in the modulation of immune response against cancer, investigation of the role of their expression as predictive biomarkers may represent a promising direction for future research.

There are several limitations in our study. First, we have not assessed the presence of novel alleles in the studied datasets. All the samples were derived from well-studied populations, and HLA genotyping was performed under the assumption that the individuals harbor HLA alleles already present in the IPD-IMGT/HLA DB. Secondly, even though we were able to identify interesting patient-specific trends relating to HLA expression and immune cell infiltration, the number of samples was too low to make statistically robust conclusions. Another limitation relates to the nature of sampling normal adjacent tissues. Comparing tumor with normal tissue samples is important for understanding the mechanisms of cancer progression and has many advantages over tumor-only approaches. Moreover, it has even been reported that paired normal samples are in general more informative on patient survival than tumors [84]. However, collecting matched normal tissues from patients is either impossible or at best challenging. In addition, there is some uncertainty regarding whether histologically normal adjacent to tumor samples are molecularly normal. Aran et *al*., have performed a comprehensive analysis of normal adjacent to tumor transcriptomes and concluded that these tissues rather present a unique intermediate state between healthy and tumor [85]. This implies that the normal adjacent tissues used in the current study may bear the neighboring tumor microenvironment signals and can’t be considered as truly ‘normal’. However, despite these potential limitations, the comparison of normal adjacent to tumor tissue and tumor can provide valuable insights into the tumor microenvironments and the interplay between tumors and nearby tissues. For example, it has been demonstrated, that the evaluation of the microenvironment in breast tumors is essential for predicting recurrence and aiding surgical strategies settings [86, 87]. It would be of great interest to see the unique HLA expression and immune cell infiltration patterns as well as those shared between normal adjacent and distal microenvironments to the tumor.

In summary, we have developed a novel method for HLA genotyping and expression estimation from RNA-seq and demonstrated that transcriptome profiling of tumor versus normal adjacent to tumor tissues can reveal interesting insights into the characterization of adjacent normal tissue and can be of value for studying the interplay between the HLA genotype expression patterns and tumor immune escape. Given the pivotal role of HLA genes in tumor cell recognition, further experiments are crucially required to validate the role of HLA expression as a novel potential biomarker in cancer immunotherapy. We believe that the methodology described here for the integrative and accurate HLA genotyping and personal HLA expression estimation may help to capture further insights into the role of HLA expression in the mechanisms of tumor progression and immune evasion and help to identify evidence of HLA expression as a biomarker in patient stratification in cancer immunotherapy.

### Supplementary Information

**Additional file 1: Table S1.** Table showing the accession numbers of the matched tumor-normal adjacent tissue pairs from the publicly available dataset GSE58135 as well as predicted HLA genotypes for classical HLA Class I genes. **Table S2.** Table showing HLA typing accuracies for OncoHLA and five tools of the benchmarking set on the GEUVADIS dataset with updated ground truth typing. **Table S3.** Table showing mean fold change in expression and *P*-values of HLA loci and B2M protein in ER+ HER2- primary tumor compared to matched normal adjacent tissues. **Table S4.** Table showing mean fold change in expression and *P*-values of HLA loci and B2M protein in TNBC primary tumor compared to matched normal adjacent tissues. **Figure S1.** Boxplots representing log-scaled allele-specific expression of HLA loci and B2M protein in two breast cancer subtypes and normal adjacent to tumor tissues. **Figure S2.** Volcano plot of differential gene expression of HLA loci and B2M protein in tumor versus matched normal adjacent tissues. **Figure S3.** Radar plots showing HLA/B2M expression in 20 ER+ tumor-normal adjacent tissue pairs from the publicly available dataset GSE58135. **Figure S4.** Radar plots showing HLA/B2M expression in 15 TNBC tumor-normal adjacent tissue pairs from the public dataset GSE58135.

## Data Availability

The publicly available data used for HLA typing performance evaluation was downloaded from: http://www.ebi.ac.uk/arrayexpress/experiments/E-GEUV-1/files/processed. The publicly available dataset containing breast cancer RNA-seq data can be found on gene expression omnibus (GEO) under accession number GSE58135.

## Abbreviations

APM: Antigen processing machinery
B2M: Beta-2 microglobulin
DB: Database
ER +: Estrogen receptor-positive
FFPE: Formaldehyde-fixed paraffin-embedded
GEUVADIS: Genetic European variation in health and disease
HLA: Human leukocyte antigen
ILP: Integer linear programming
MHC: Major histocompatibility complex
ML: Machine-learning
NGS: Next-generation sequencing
NK: Natural Killer
RIN: RNA integrity number
SBT: Sequence-based typing
SSOP: Sequence-specific oligonucleotide probe
SSP: Sequence-specific primer
TPM: Transcript per million
TNBS: Triple-negative breast cancer
WES: Whole exome sequencing
XML: Extensive markup language
