# Supplementary material for: Integrative HLA typing of tumor and adjacent normal tissue can reveal insights into the tumor immune response
Source: BMC Med Genomics. 2024 Jan 27;17:37. doi: 10.1186/s12920-024-01808-8 (PMC10821267; doi:10.1186/s12920-024-01808-8)
Supplement: Supplementary file 1 — Additional file 1: Table S1. Table showing the accession numbers of the matched tumor-normal adjacent tissue pairs from the publicly available dataset GSE58135 as well as predicted HLA genotypes for classical HLA Class I genes. Table S2. Table showing HLA typing accuracies for OncoHLA and five tools of the benchmarking set on the GEUVADIS dataset with updated ground truth typing. Table S3. Table showing mean fold change in expression and P-values of HLA loci and B2M protein in ER+ HER2- primary tumor compared to matched normal adjacent tissues. Table S4. Table showing mean fold change in expression and P-values of HLA loci and B2M protein in TNBC primary tumor compared to matched normal adjacent tissues. Figure S1. Boxplots representing log-scaled allele-specific expression of HLA loci and B2M protein in two breast cancer subtypes and normal adjacent to tumor tissues. Figure S2. Volcano plot of differential gene expression of HLA loci and B2M protein in tumor versus matched normal adjacent tissues. Figure S3. Radar plots showing HLA/B2M expression in 20 ER+ tumor-normal adjacent tissue pairs from the publicly available dataset GSE58135. Figure S4. Radar plots showing HLA/B2M expression in 15 TNBC tumor-normal adjacent tissue pairs from the public dataset GSE58135. [file 12920_2024_1808_MOESM1_ESM.docx]

Integrative HLA typing of tumor and adjacent normal tissue can reveal insights into the tumor immune response

**Supplementary Materials:**

**Table S1:** Table showing the accession numbers of the matched tumor-normal adjacent tissue pairs from the publicly available dataset GSE58135 as well as predicted HLA genotypes for classical HLA Class I genes.

**Table S2:** Table showing HLA typing accuracies for OncoHLA and five tools of the benchmarking set on the GEUVADIS dataset with updated ground truth typing.

**Table S3:** Table showing mean fold change in expression and *P*-values of HLA loci and B2M protein in ER+ HER2- primary tumor compared to matched normal adjacent tissues.

**Table S4:** Table showing mean fold change in expression and *P*-values of HLA loci and B2M protein in TNBC primary tumor compared to matched normal adjacent tissues.

**Figure S1:** Boxplots representing log-scaled allele-specific expression of HLA loci and B2M protein in two breast cancer subtypes and normal adjacent to tumor tissues.

**Figure S2:** Volcano plot of differential gene expression of HLA loci and B2M protein in tumor versus matched normal adjacent tissues.

**Figure S3:** Radar plots showing HLA/B2M expression in 20 ER+ tumor-normal adjacent tissue pairs from the publicly available dataset GSE58135.

**Figure S4:** Radar plots showing HLA/B2M expression in 15 TNBC tumor-normal adjacent tissue pairs from the public dataset GSE58135.

| **Breast cancer subtype** | **Normal ID** | **Normal HLA genotypes** | **Tumor ID** | **Tumor HLA genotypes** |
| --- | --- | --- | --- | --- |
| **ER+** | SRR1313203 | A*03:01:01  A*74:01:01  B*07:06:01  B*58:01:01  C*03:02:02  C*07:02:01 | SRR1313126 | A*03:01:01  A*74:01:01  B*07:06:01  B*58:01:01  C*03:02:02  C*07:02:01 |
|  | SRR1313202 | A*03:01:01  A*31:01:02  B*07:02:01  B*14:02:01  C*07:02:01  C*08:02:01 | SRR1313107 | A*03:01:01  A*31:01:02  B*07:02:01  B*14:02:01  C*07:02:01  C*08:02:01 |
|  | SRR1313201 | A*02:01:01  B*07:02:01  B*15:01:01  C*04:01:01  C*05:01:01 | SRR1313129 | A*02:01:01  B*07:02:01  B*15:01:01  C*04:01:01  C*05:01:01 |
|  | SRR1313200 | A*33:01:01  A*34:02:01  B*35:01:01  B*40:02:01  C*02:02:02  C*04:01:01 | SRR1313128 | A*33:01:01  A*34:02:01  B*35:01:01  B*40:02:01  C*02:02:02  C*04:01:01 |
|  | SRR1313199 | A*01:01:01  A*24:02:01  B*07:02:01  B*57:01:01  C*06:02:01  C*07:02:01 | SRR1313127 | A*01:01:01  A*24:02:01  B*07:02:01  B*57:01:01  C*06:02:01  C*07:02:01 |
|  | SRR1313198 | A*01:01:01  A*02:01:01  B*08:01:01  B*15:01:01  C*03:04:01  C*07:01:01 | SRR1313123 | A*01:01:01  A*02:01:01  B*08:01:01  B*15:01:01  C*03:04:01  C*07:01:01 |
|  | SRR1313195 | A*02:01:01  A*25:01:01  B*18:01:01  B*27:05:02  C*02:02:02  C*12:03:01 | SRR1313131 | A*02:01:01  A*25:01:01  B*18:01:01  B*27:05:02  C*02:02:02  C*12:03:01 |
|  | SRR1313194 | A*02:01:01  A*03:01:01  B*40:01:02  B*51:08:01  C*03:04:01  C*16:02:01 | SRR1313113 | A*02:01:01  A*03:01:01  B*40:01:02  B*51:08:01  C*03:04:01  C*16:02:01 |
|  | SRR1313193 | A*02:01:01  A*32:01:01  B*39:24:01  B*44:02:01  C*05:01:01  C*07:01:01 | SRR1313112 | A*02:01:01  A*32:01:01  B*39:24:01  B*44:02:01  C*05:01:01  C*07:01:01 |
|  | SRR1313192 | A*02:01:01  A*29:01:01  B*07:05:01  B*40:01:02  C*03:04:01  C*15:05:02 | SRR1313111 | A*02:01:01  A*29:01:01  B*07:05:01  B*40:01:02  C*03:04:01  C*15:05:02 |
|  | SRR1313191 | A*24:02:01  A*31:01:02  B*40:01:02  B*44:02:01  C*03:04:01  C*05:01:01 | SRR1313110 | A*24:02:01  A*31:01:02  B*40:01:02  B*44:02:01  C*03:04:01  C*05:01:01 |
|  | SRR1313190 | A*02:01:01  A*03:01:01  B*15:01:01  B*15:01:01  C*03:03:01  C*03:04:01 | SRR1313109 | A*02:01:01  A*03:01:01  B*15:01:01  B*15:01:01  C*03:03:01  C*03:04:01 |
|  | SRR1313187 | A*02:01:01  A*29:02:01  B*49:01:01  B*58:01:01  C*07:01:01  C*07:18:01 | SRR1313105 | A*02:01:01  A*29:02:01  B*49:01:01  B*58:01:01  C*07:01:01  C*07:18:01 |
|  | SRR1313184 | A*02:01:01  A*02:01:01  B*44:02:01  B*44:03:01  C*04:01:01  C*05:01:01 | SRR1313102 | A*02:01:01  A*02:01:01  B*44:02:01  B*44:03:01  C*04:01:01  C*05:01:01 |
|  | SRR1313182 | A*02:01:01  A*24:02:01  B*08:01:01  B*40:01:02  C*03:04:01  C*07:01:01 | SRR1313130 | A*02:01:01  A*24:02:01  B*08:01:01  B*40:01:02  C*03:04:01  C*07:01:01 |
|  | SRR1313181 | A*02:01:01  A*03:01:01  B*27:05:02  B*51:01:01  C*02:02:02  C*14:02:01 | SRR1313098 | A*02:01:01  A*03:01:01  B*27:05:02  B*51:01:01  C*02:02:02  C*14:02:01 |
|  | SRR1313177 | A*02:01:01  A*24:02:01  B*35:02:01  B*57:01:01  C*04:01:01  C*06:02:01 | SRR1313093 | A*02:01:01  A*24:02:01  B*35:02:01  B*57:01:01  C*04:01:01  C*06:02:01 |
|  | SRR1313176 | A*03:01:01  A*03:01:01  B*07:02:01  B*55:01:01  C*03:03:01  C*07:02:01 | SRR1313092 | A*03:01:01  A*03:01:01  B*07:02:01  B*55:01:01  C*03:03:01  C*07:02:01 |
|  | SRR1313175 | A*01:01:01  A*25:01:01  B*07:02:01  B*40:01:02  C*03:04:01  C*07:02:01 | SRR1313091 | A*01:01:01  A*25:01:01  B*07:02:01  B*40:01:02  C*03:04:01  C*07:02:01 |
|  | SRR1313174 | A*03:01:01  A*11:01:01  B*27:05:02  B*57:01:01  C*01:02:01  C*06:02:01 | SRR1313090 | A*03:01:01  A*11:01:01  B*27:05:02  B*57:01:01  C*01:02:01  C*06:02:01 |
| **TNBC** | SRR1313225 | A*02:01:01  A*31:01:02  B*08:01:01  B*40:01:02  C*03:04:01  C*07:01:01 | SRR1313167 | A*02:01:01  A*31:01:02  B*08:01:01  B*40:01:02  C*03:04:01  C*07:01:01 |
|  | SRR1313223 | A*02:01:01  A*02:01:01  B*15:01:01  B*37:01:01  C*03:03:01  C*06:02:01 | SRR1313171 | A*02:01:01  A*02:01:01  B*15:01:01  B*37:01:01  C*03:03:01  C*06:02:01 |
|  | SRR1313222 | A*11:01:01  A*68:01:02  B*41:02:01  B*44:02:01  C*07:04:01  C*17:03:01 | SRR1313161 | A*11:01:01  A*68:01:02  B*41:02:01  B*44:02:01  C*07:04:01  C*17:03:01 |
|  | SRR1313221 | A*02:02:01  A*33:01:01  B*14:02:01  B*41:01:01  C*08:02:01  C*17:01:01 | SRR1313149 | A*02:02:01  A*33:01:01  B*14:02:01  B*41:01:01  C*08:02:01  C*17:01:01 |
|  | SRR1313220 | A*01:01:01  A*24:02:01  B*08:01:01  B*35:01:01  C*04:01:01  C*07:01:01 | SRR1313168 | A*01:01:01  A*24:02:01  B*08:01:01  B*35:01:01  C*04:01:01  C*07:01:01 |
|  | SRR1313218 | A*01:01:01  A*03:01:01  B*08:01:01  B*15:01:01  C*03:03:01  C*07:01:01 | SRR1313135 | A*01:01:01  A*03:01:01  B*08:01:01  B*15:01:01  C*03:03:01  C*07:01:01 |
|  | SRR1313217 | A*02:01:01  A*34:02:01  B*35:01:01  B*35:01:01  C*04:01:01  C*06:02:01 | SRR1313132 | A*02:01:01  A*34:02:01  B*35:01:01  B*35:01:01  C*04:01:01  C*06:02:01 |
|  | SRR1313216 | A*02:01:01  A*02:01:01  B*07:02:01  B*15:01:01  C*01:02:01  C*07:02:01 | SRR1313173 | A*02:01:01  A*02:01:01  B*07:02:01  B*15:01:01  C*01:02:01  C*07:02:01 |
|  | SRR1313215 | A*03:01:01  A*33:01:01  B*15:03:01  B*78:01:01  C*16:01:01 C*16:01:01 | SRR1313150 | A*03:01:01  A*33:01:01  B*15:03:01  B*78:01:01  C*16:01:01 C*16:01:01 |
|  | SRR1313214 | A*30:02:01  A*66:01:01  B*14:01:01  B*15:03:01  C*08:02:01  C*12:03:01 | SRR1313166 | A*30:02:01  A*66:01:01  B*14:01:01  B*15:03:01  C*08:02:01  C*12:03:01 |
|  | SRR1313213 | A*03:01:01  A*03:01:01  B*35:01:01  B*81:01:01  C*04:01:01  C*08:04:01 | SRR1313163 | A*03:01:01  A*03:01:01  B*35:01:01  B*81:01:01  C*04:01:01  C*08:04:01 |
|  | SRR1313212 | A*03:01:01  A*03:01:01  B*14:01:01  B*40:02:01  C*02:02:02  C*08:02:01 | SRR1313153 | A*03:01:01  A*03:01:01  B*14:01:01  B*40:02:01  C*02:02:02  C*08:02:01 |
|  | SRR1313211 | A*02:01:01  A*25:01:01  B*18:01:01  B*44:02:01  C*05:01:01  C*12:03:01 | SRR1313162 | A*02:01:01  A*25:01:01  B*18:01:01  B*44:02:01  C*05:01:01  C*12:03:01 |
|  | SRR1313210 | A*01:01:01  A*02:01:01  B*08:01:01  B*51:01:01  C*07:01:01  C*15:02:01 | SRR1313157 | A*01:01:01  A*02:01:01  B*08:01:01  B*51:01:01  C*07:01:01  C*15:02:01 |
|  | SRR1313209 | A*01:01:01  A*03:01:01  B*08:01:01  B*15:03:01  C*02:10:01  C*07:01:01 | SRR1313159 | A*01:01:01  A*03:01:01  B*08:01:01  B*15:03:01  C*02:10:01  C*07:01:01 |

**Table S1.** Table showing the accession numbers of matched tumor–normal adjacent tissue pairs from the publicly available dataset GSE58135 as well as predicted HLA genotypes for classical HLA Class I genes.

|  | **Class I** | | | **Class II** | |
| --- | --- | --- | --- | --- | --- |
|  | A | B | C | DRB1 | DQB1 |
| **OptiType** | 99.7 | 99.4 | 100 | - | - |
| **Seq2HLA** | 98.2 | 95 | 94.7 | 92.3 | 79.5 |
| **HLAProfiler** | 95.7 | 86.3 | 87.4 | 92.2 | 92.3 |
| **HLAPers** | 92.5 | 97.1 | 47.2 | 97.9 | 95.7 |
| **ArcasHLA** | 98 | 99 | 95.3 | 98.2 | 92.3 |
| **OncoHLA** | 99.2 | 99.3 | 99.2 | 99.9 | 99.7 |

**Table S2.** Table showing accuracy for OncoHLA and five tools of the benchmarking set on the GEUVADIS dataset with updated ground truth typing. Values in the table correspond to accuracy in percentage at two fields of resolution.

| **HLA locus** | **log2FC** | ***P*-value** | **adjusted *P*-value** |
| --- | --- | --- | --- |
| **B2M** | -0.7737818930714084 | 0.0029275712162333267 | 0.03513085459479992 |
| **A** | -0.296273006709292 | 0.4787398367615241 | 1.0 |
| **B** | -0.5756790332343318 | 0.05438970471202653 | 0.6526764565443184 |
| **C** | -0.45335897449905893 | 0.022351041959343565 | 0.2682125035121228 |
| **E** | -1.8702066915006608 | 4.1174508691455e-08 | 4.9409410429746e-07 |
| **F** | 0.2284937588048983 | 0.3894960477845295 | 1.0 |
| **G** | -0.5004694114970667 | 0.21884284079083005 | 1.0 |
| **DPA1** | -0.9636694945945621 | 0.002873920752741732 | 0.034487049032900785 |
| **DPB1** | -0.7384165234451645 | 0.013520309408006526 | 0.1622437128960783 |
| **DQA1** | -0.6996779950653735 | 0.1340808627395147 | 1.0 |
| **DQB1** | -0.34160079125960063 | 0.37614606549598006 | 1.0 |
| **DRB1** | -0.8425575887599539 | 0.008913600990792192 | 0.1069632118895063 |

**Table S3.** Table showing mean fold change in expression and *P*-values of HLA loci and B2M protein in ER+ HER2- primary tumor compared to matched normal adjacent tissues. *P*-values were adjusted with a Bonferroni correction for multiple comparisons.

| **HLA locus** | **log2FC** | **P-value** | **adjusted P-value** |
| --- | --- | --- | --- |
| **B2M** | -0.4957377528752396 | 0.07678798196672876 | 0.9214557836007451 |
| **A** | -0.06566911869361647 | 0.8449667964593222 | 1.0 |
| **B** | -0.0361011129446549 | 0.9153072822484678 | 1.0 |
| **C** | -0.21295224633862908 | 0.48008979497996385 | 1.0 |
| **E** | -1.7838045546895547 | 1.4199696174443943e-06 | 1.7039635409332732e-05 |
| **F** | 1.224506354766734 | 0.026911523400456883 | 0.3229382808054826 |
| **G** | 0.007120387647922666 | 0.9845787261949377 | 1.0 |
| **DPA1** | -0.8519585777763803 | 0.02139906541405545 | 0.2567887849686654 |
| **DPB1** | -0.6624707317616529 | 0.041334689300747574 | 0.4960162716089709 |
| **DQA1** | -0.04541862686026121 | 0.9209967525008735 | 1.0 |
| **DQB1** | -0.095749774946424 | 0.8159971898834522 | 1.0 |
| **DRB1** | -0.682751366920229 | 0.06994865917401638 | 0.8393839100881966 |

**Table S4.** Table showing mean fold change in expression and *P*-values of HLA loci and B2M protein in TNBC primary tumor compared to matched normal adjacent tissues. *P*-values were adjusted with a Bonferroni correction for multiple comparisons.

**Figure S1.** Boxplots (including median, 95 % confidence intervals, 1^st^ and 3^rd^ quartiles, maximum and minimum) representing log-scaled allele-specific expression of classical and non-classical HLA loci and B2M protein in two breast cancer subtypes (ER+ HER2- breast cancer subtype in green and TNBC breast cancer subtype in blue) and normal adjacent to tumor tissues (in gray). **A**. ER+ HER2- primary tumor vs. matched normal adjacent tissue. **B**. TNBC primary tumor vs. matched normal adjacent tissue.

**Figure S2.** Volcano plot of differential gene expression of HLA loci and B2M protein in tumor versus matched normal adjacent tissues. Each point represents a gene. The expression difference is considered significant for a log2 fold change of <=1 and >=1 and for a *P*-value of <= 0.05. **A**. ER+ HER2- primary tumor vs. matched normal adjacent tissue. **B**. TNBC primary tumor vs. matched normal adjacent tissue.


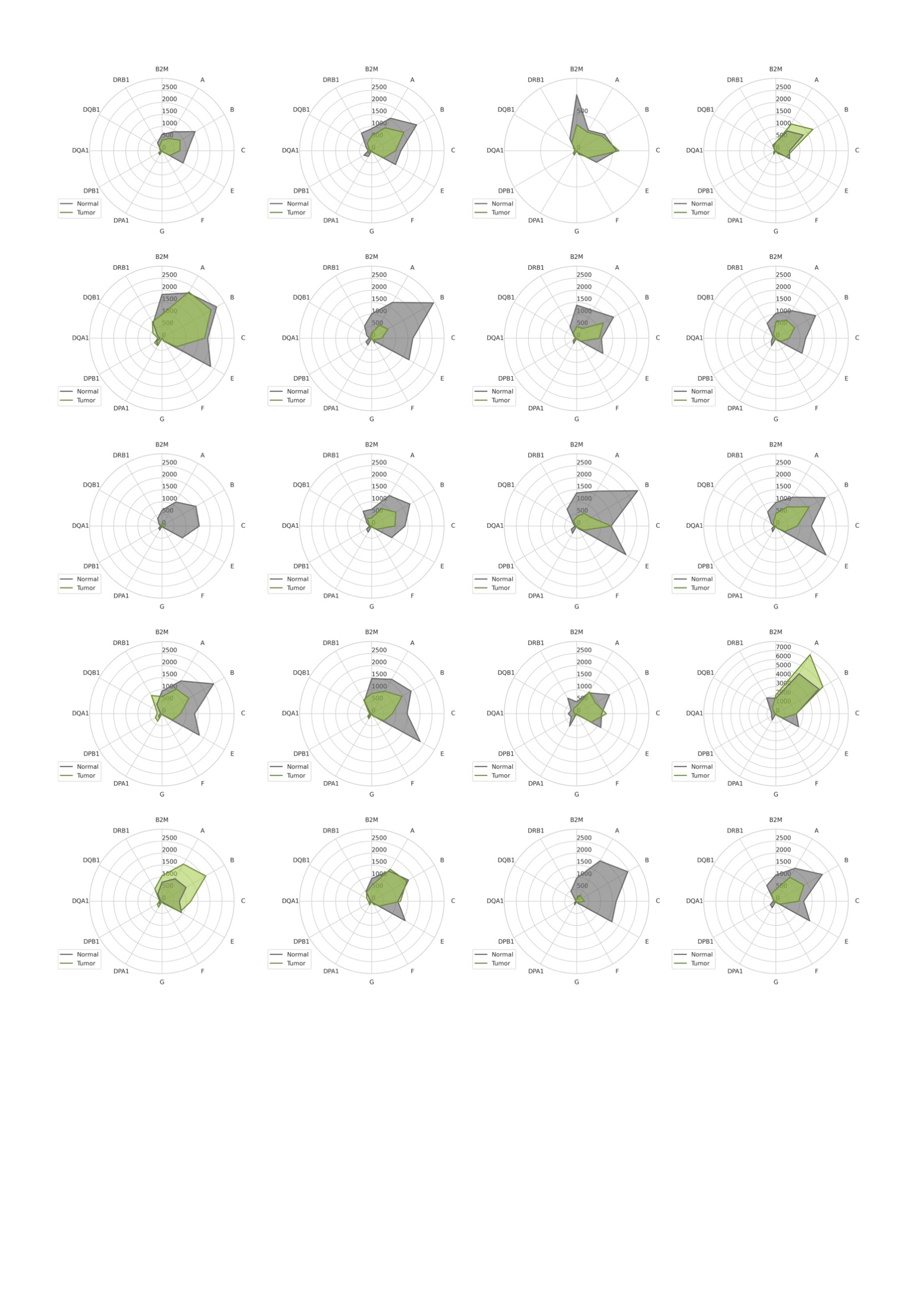


**Figure S3.** Radar plots showing HLA/B2M expression in 20 ER+ tumor-normal adjacent tissue pairs from the public dataset GSE58135.


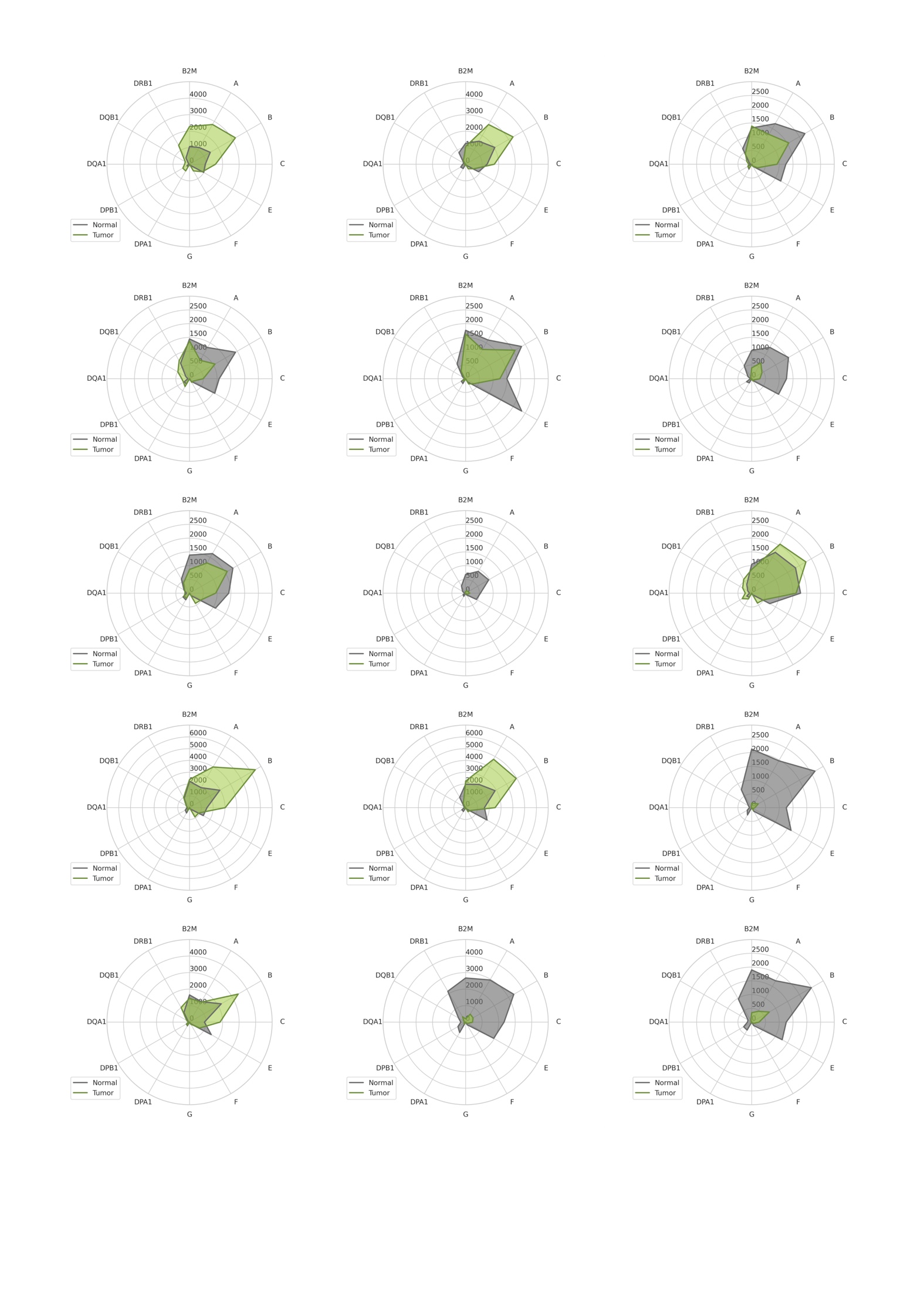


**Figure S4.** Radar plots showing HLA/B2M expression in 15 TNBC tumor-normal adjacent tissue pairs from the public dataset GSE58135.
